# Structure of the mammalian ribosomal pre-termination complex associated with eRF1•eRF3•GDPNP

**DOI:** 10.1093/nar/gkt1279

**Published:** 2013-12-11

**Authors:** Amédée des Georges, Yaser Hashem, Anett Unbehaun, Robert A. Grassucci, Derek Taylor, Christopher U. T. Hellen, Tatyana V. Pestova, Joachim Frank

**Affiliations:** ^1^Howard Hughes Medical Institute, Chevy Chase, MD, USA, ^2^Department of Biochemistry and Molecular Biophysics, Columbia University, New York, NY, USA, ^3^Department of Cell Biology, SUNY Downstate Medical Center, Brooklyn, NY, USA, ^4^Department of Pharmacology, Case Western Reserve University, Cleveland, OH, USA and ^5^Department of Biological Sciences, Columbia University, New York, NY, USA

## Abstract

Eukaryotic translation termination results from the complex functional interplay between two release factors, eRF1 and eRF3, in which GTP hydrolysis by eRF3 couples codon recognition with peptidyl-tRNA hydrolysis by eRF1. Here, we present a cryo-electron microscopy structure of pre-termination complexes associated with eRF1•eRF3•GDPNP at 9.7 -Å resolution, which corresponds to the initial pre-GTP hydrolysis stage of factor attachment and stop codon recognition. It reveals the ribosomal positions of eRFs and provides insights into the mechanisms of stop codon recognition and triggering of eRF3’s GTPase activity.

## INTRODUCTION

Termination occurs when a stop codon enters the ribosomal A site and consists of stop codon recognition followed by peptide release, which involves the nucleophilic attack of a water molecule on the P-site peptidyl-tRNA in the ribosomal peptidyl transferase center (PTC). Eukaryotic termination is mediated by two directly interacting release factors: eRF1, which is responsible for stop codon recognition and triggering peptide release, and eRF3, a GTPase that strongly stimulates peptide release by eRF1 in a GTP-dependent manner (1–3). eRF1, in turn, stabilizes binding of GTP to eRF3 so that they form a stable ternary complex (4,5), and is required for eRF3’s ribosome-dependent GTPase activity (6). eRF1 has omnipotent decoding capacity and recognizes all three stop codons.

It comprises N-terminal (N), middle (M) and C-terminal (C) domains (7). The rigid core of domain C contains a flexible insertion forming a mini-domain (8). Domain N is involved in stop codon recognition. Although the mechanism by which eRF1 responds to all three stop codons is not clear, extensive mutational and genetic analyses identified the essential role in this process of GTS_31–__33_ (human numbering), TASNIKS_58–__64_ and YxCxxxF_125–__131_ motifs located at the apex of the N-domain (7,9–18). eRF1’s domain M contains the universal GGQ loop, whose placement into the PTC causes rearrangement of rRNA, allowing a water molecule to enter and induce peptide release (1,7,19–22).

eRF3 consists of the essential C-terminal region comprising GTP-binding (G) domain and β-barrel domains 2 and 3, which are homologous to elongation factors EF-Tu and eEF1A (23), and a non-conserved N-terminal region that is not essential for termination (24). eRF1’s C and M domains interact with eRF3 (15,25). eRF1 and eRF3 bind to the pre-termination complexes (pre-TCs) as an eRF1•eRF3•GTP ternary complex, but peptide release does not occur until eRF3 hydrolyzes GTP. GTP hydrolysis releases eRF1’s domain M from eRF3, which enables its GGQ loop to enter the PTC and trigger peptide release.

Although the structures of individual eRFs have been determined (7,15,23), the structural basis for key steps in termination, such as stop codon recognition or triggering of eRF3’s GTPase activity, remains unresolved. Our previous cryo-EM structure of the eRF1•eRF3•GDPNP-bound pre-TC had a resolution of 18 Å (26), which did not allow accurate modeling of the ribosome-bound factors and therefore could not address such questions. The advent of a more powerful classification algorithm, RELION (27), prompted us to re-examine the original data set (26), resulting in a much higher resolution (9.7 Å), which enabled us to propose a more detailed model of the interactions pivotal for peptide release.

## MATERIALS AND METHODS

### Data processing

**Figure 1.**
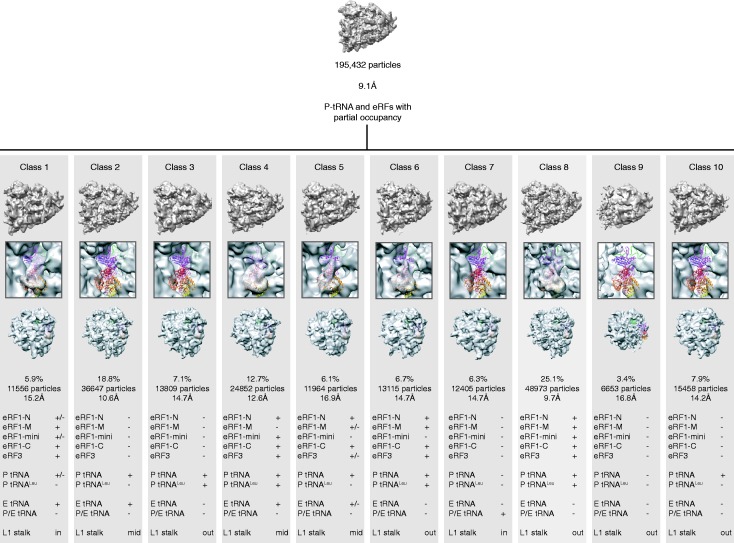
Unsupervised 3D classification. The 195 432 particles were first refined with RELION (27) to give a 9.1-Å ribosome reconstruction having low occupancy for the factors and tRNAs (top)**.** The particles were then classified with RELION (27) starting with 10 seeds. The 10 resulting maps of the ribosome (bottom) are shown from the P stalk side (top vignette), with a close-up on the eRF1-eRF3 binding site (middle vignette), and from the 40S solvent side (bottom vignette). Bottom table: strength of the electron density for each domain of eRF1, eRF3 and tRNAs, as well as position of the L1 stalk.

Mammalian pre-TCs were assembled *in vitro* on a derivative of β-globin messenger RNA (mRNA) encoding MVHL tetrapeptide followed by a UAA stop codon, using purified rabbit 40S and 60S ribosomal subunits, initiation (2,3,1,1A,4A,4B,4G,5 and 5B) and elongation (1A and 2) factors and aminoacylated tRNAs (26). eRF1•eRF3•GDPNP-bound pre-TCs were formed with full-length eRF1 and eRF3 lacking the non-essential N-terminal 138aa (26). A cryo-EM data set of the eRF1•eRF3•GDPNP-bound pre-TCs comprising 195 432 particles (26) was aligned and refined using the RELION autorefine procedure (27). It resulted in a 9.1-Å structure [gold standard Fourier shell correlation (FSC) = 0.143] showing fragmented densities in the intersubunit space, indicative of heterogeneity. The data set was then subjected to RELION classification (28) starting with 10 classes (k = 10). Of the 10 classes, class 8 (Figure 1) was well populated (48 973 particles, 25.1%), with well-defined additional masses of density in the intersubunit space corresponding to eRF1, eRF3 and P-site tRNA as previously described (26). The particles from this class were isolated, and the structure was first refined with RELION autorefine procedure. This yielded a structure at 9.7 Å (gold standard FSC = 0.143), but the density exhibited strong stretching artifacts when filtered at the measured resolution, probably due to preferred orientations of the particles on the cryo-EM grid. The alignment parameters were further refined using RELION classification with k = 1 and a T factor of four for three iterations at an angular spacing of 1.8°, followed by three additional iterations at 0.9°. The resulting reconstruction displayed the same resolution of 9.7 Å (FSC = 0.143), but the quality of the structure was very much improved and the stretching artifacts were no longer visible. The statistics of alignment precision estimated by RELION were also significantly improved [improvement in terms of overall accuracy of rotations, 41%; overall accuracy of translations, 43% and average Pmax at 1.8° angular step, 180% (Supplementary Table S1)].

### Modeling of eRF1, eRF3, mRNA and P-site tRNA^Leu^

The cryo-EM map segmentation was performed using the Segger (29) plug-in in UCSF Chimera (30). Models of human eRF1 and eRF3 were built based on several experimental structures. Atomic coordinates of eRF1 and eRF3 domains 2 and 3 were taken from their crystal structure (15) (PDBID: 3E1Y), and the C-terminal mini-domain structure of eRF1 (residues 326–373) was modeled based on its nuclear magnetic resonance (NMR) structure (8) (PDBID: 2KTU). Most of eRF3 G-domain (residues 317–439, 207–216) was modeled by homology based on the crystal structure of *Saccharomyces pombe* eRF3•GDP (23) (PDBID: 1R5B) because of its significantly higher resolution over the *S. pombe* eRF3•GMPPNP crystal structure (23) (2.35 versus 3.20 Å, respectively). Only the GTP-binding pocket of the G-domain (residues 217–231) was modeled based on the crystal structure of eRF3•GMPPNP (23) (PDBID: 1R5O). Missing residues and the switch region (residues 232–316) in eRF3 G-domain were modeled by homology based on the archeal elongation factor 1α (aEF1α) taken from the eRF1•GTP-bound aEF1α crystal structure (31) (PDBID: 3VMF). The arrangement between eRF3’s domains II and III and G-domain was derived from the latter crystal structure as well. The P-site tRNA^Leu^ was modeled from its crystal structure in complex with leucyl-tRNA synthetase from *Pyrococcus horikoshii* (32) (PDBID: 1WZ2). The mRNA was modeled based on the mRNA of the crystal structure of the *Thermus thermophilus* 70S ribosome bound with the Q253P mutant form of release factor 2 (22) (PDBID: 4KFK). All homology modeling was done using SWISS-MODEL (33,34).

### Molecular dynamics flexible fitting

The derived model was first rigid body-fitted into its corresponding segmented cryo-EM density map using UCSF Chimera (30). Then, using the molecular graphics software VMD (35), the system was prepared for molecular dynamics flexible fitting (MDFF) (36) in explicit solvent, a procedure which applies the cryo-EM map as an additional potential to the system—in this study the eRF1•eRF3•tRNA•mRNA cryo-EM segmented map—thus comprising only the molecules to be simulated: eRF1, eRF3, mRNA and P-site tRNA^Leu^. The MDFF system was embedded in a box of TIP3P water molecules with an extra 12-Å padding in each direction. The system was neutralized by potassium ions, and an excess of ∼0.2 M KCl was added. The simulated system was prepared using CHARMM force field parameters [Combined CHARMM All-hydrogen topology file for CHARMM22 proteins and CHARMM27 lipids (37)]. The system was energy-minimized in 600 steps in the molecular dynamics simulation package NAMD (38). After minimization, the fitting trajectories were run for 400 ps, once the root-mean-square deviation and cross-correlation coefficient had stabilized.

## RESULTS AND DISCUSSION

### Structure determination of the mammalian eRF1•eRF3•GDPNP-bound pre-TC

**Figure 2.**
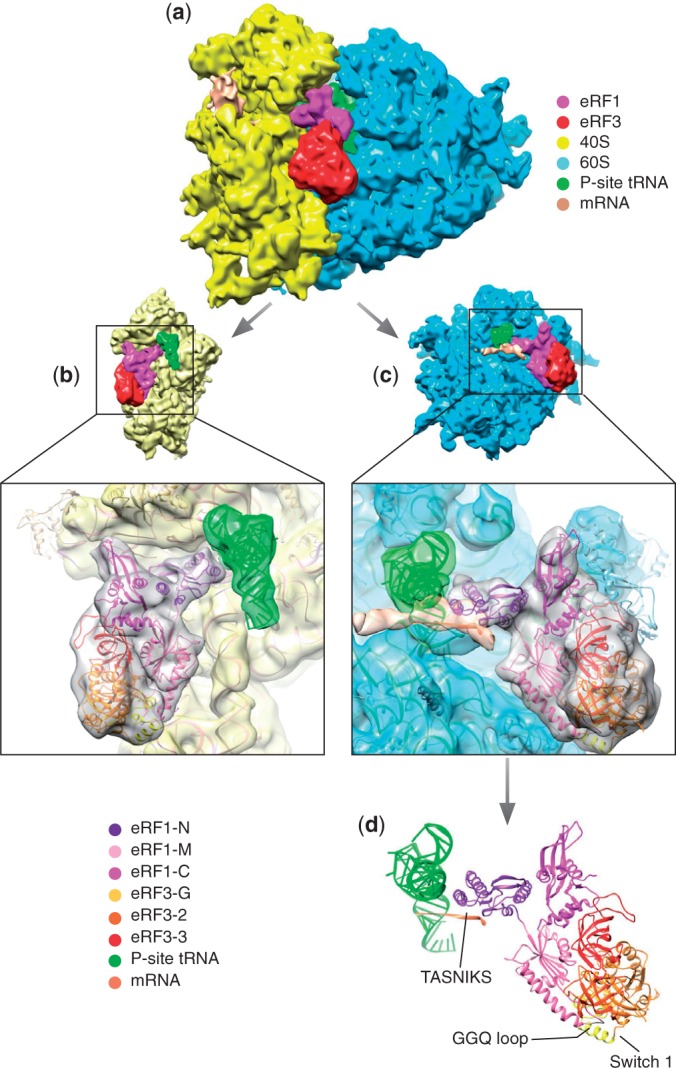
Overview of the complex. (**a**) Overview of the map showing the 40S (yellow), 60S (blue), eRF1 (Magenta), eRF3 (red), P-site tRNA (green) and mRNA (coral). (**b**) Atomic model fitted into its density viewed from the 60S side with the 60S density removed. (**c**) Atomic model fitted into its density viewed from the 40S side with the 40S density removed. (**d**) Atomic model in the same orientation as in c, showing eRF1, eRF3, the P-site tRNA and mRNA path.

Classification of the cryo-EM data set comprising 195 432 particles of the mammalian eRF1•eRF3•GDPNP-bound pre-TC formed on mRNA encoding MVHL tetrapeptide, followed by a UAA stop codon, yielded 10 classes with different factor and tRNA occupancies (Figure 1). One of these classes (25% of the particles) had P-site tRNA along with well-defined eRF1 and eRF3 densities. The characteristic long variable loop of tRNA^Leu^ was apparent in the P-site tRNA, indicating that it was correctly programmed with an A-site stop codon. The eRF1•eRF3•GDPNP-bound pre-TC class yielded a 9.7-Å reconstruction (Figure 2a), which allowed precise docking of each factor's domains. eRF1 and eRF3 were modeled (Figures 2b–d) based on the crystal structures of eRF1, eRF3, eRF1-eRF3_2-3_ and aRF1-aEF1α (7,15,23,31) and the NMR structure of eRF1’s C-domain (8). The model was refined using MDFF (36) with secondary structure constraints.

### Overview of the complex

The position and conformation of ribosome-bound eRF1/eRF3 are similar to those of aa-tRNA/EF-Tu (39) and the eRF1/eRF3 paralogs Dom34/Hbs1 (40) involved in the dissociation of stalled elongation complexes (41,42), with eRF1’s domain N binding to the decoding center, and the rigid core and mini-domain of eRF1’s domain C forming a bridge between the P stalk and the beak of the 40S subunit. eRF1 interacts with eRF3’s domain 3 via its domain C, whereas eRF1’s extended domain M containing the GGQ motif is tucked between eRF3’s domains 2 and G. eRF3 is bound to the universal GTPase-associated center (GAC) of the ribosome, between the sarcin–ricin loop (SRL) on the 60S subunit, and helices (h) 5 and 14 of 18S rRNA on the 40S subunit. No additional conformational changes were observed on the ribosome at the improved resolution, compared with those reported previously (26): it is in a non-rotated state, the L1 stalk in the open position (Figure 1) and the P stalk base shifted inward. However, what was previously interpreted as the result of a ribosomal rearrangement at the mRNA entrance (26) now appears more likely to be an mRNA bundle (Figure 2 and Supplementary Figure S1).

### Interaction between eRF1-N and the stop codon

**Figure 3.**
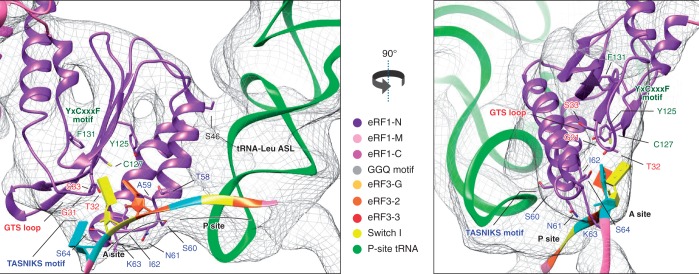
Close-up of the eRF1 N-domain and mRNA showing the TASNIKS amino-acids (blue), GTS loop (red) and YxCxxxF motif (green), and their positions relative to the approximate location of the stop codon bases represented as slabs. Codon positions are indicated in orange: first position, yellow: second position and blue: third position.

eRF1’s N-domain reaches deep into the decoding center (Figure 2), establishing multiple contacts with the 40S subunit, including h18, h30, h31, h34 and h44 of 18S rRNA and ribosomal proteins rpS30e and rpS31e. R65/R68 in the long α3 helix are in proximity to nucleotides 1330–1331 of h34, consistent with these residues’ strong influence on eRF1’s ribosomal binding (11). In our structure, mRNA occupies its normal position in the decoding center, and eRF1’s N-domain interacts directly with the stop codon (Figure 3), primarily via the TASNIKS_58–__64_ motif, consistent with ultraviolet cross-linking of the stop codon to this element (10). The positions of the mRNA bases and amino acid side-chains cannot be determined precisely at the present resolution. However, the observed proximity of particular residues and bases in the fitted structures makes it likely that they interact with one another. The TASN residues appear to be close to the first position of the stop codon, I to the second and KS to the third. The proximity of K to the nucleotide immediately downstream of the stop codon could potentially contribute to the mechanism by which this base influences termination efficiency (43,44). In addition to TASNIKS, three residues from GTS_31–__33_ and YxCxxxF_125–__131_ motifs are also positioned within interacting distance of the stop codon: G31 and T32 are close to the third and second base, respectively, and C127 to the bases in positions 1 and 2. The positions of stop codon nucleotides relative to these three residues are consistent with the experimentally determined influence of T32 and C127 on the specificity of recognition of the nucleotide in the second position of the stop codon (14,15,17). Y125 and F131 are farther away, and their influence on termination (9,12,15,17) may therefore reflect roles in stabilizing the domain structure. Additional contacts with the pre-TC may be important for proper placement of the N-domain into the decoding site. Thus, the β-sheet supporting the GTS and YCF motifs interacts with H69 of 28S rRNA, whereas the continuous density between helix α2 and the P-site tRNA (which is particularly strong between S46 at the tip of α2 and nucleotide 30 of the tRNA^Leu^ anticodon stem-loop) indicates likely contact between eRF1 and P-site tRNA. Functional interaction between them has been proposed, and preferential binding to specific tRNAs could contribute to the observed bias for certain codons preceding stop codons (45,46). Interestingly, the characteristic long variable loop of the P-site tRNA^Leu^ is also in proximity to the highly conserved P-site loop of rpL11 on the 60S subunit and to h30 and rpS18 of the 40S subunit (Supplementary Figure S2). The P-site loop of rpL11 (L5 in bacteria) contacts the T-loop of tRNA in the P/P, as well as in neighboring intermediate states, and was therefore proposed to escort tRNA through the P site during translocation (47–50). Notably, residues in the P-site loop, which are seen in proximity to the variable loop of tRNA^Leu^ in the present reconstruction, were shown to play a key role in binding of the P-site tRNA (51). The potential strengthening of the contact with the P-site loop of L11 and additional specific contacts with h30 and rpS18 may contribute to the known higher affinity of tRNA^Leu^ to the P/P state, accounting for the increased stability of ribosomal complexes containing P-site tRNA^Leu^ after peptide release (52).

**Figure 4.**
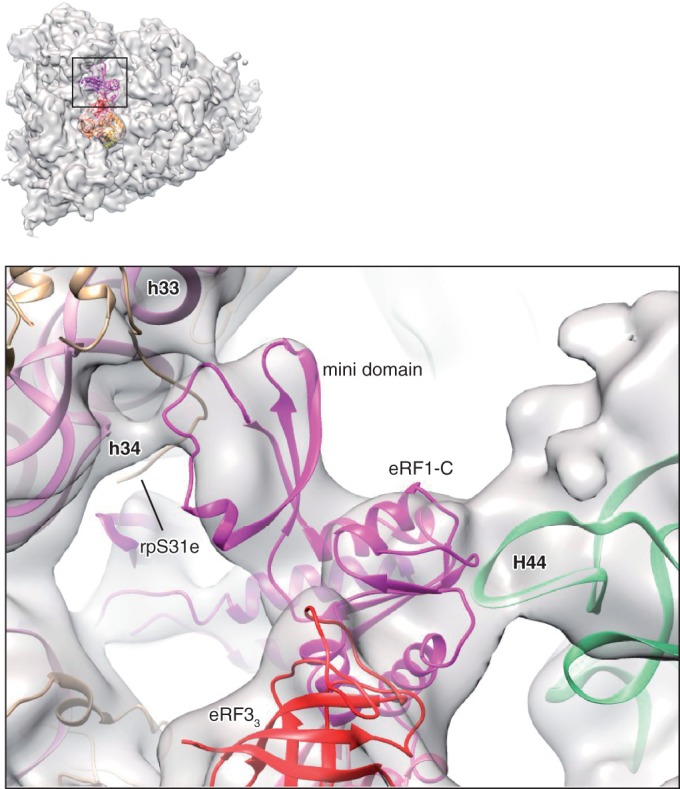
Close-up of eRF1-C and the mini-domain, showing the interaction of eRF1-C with H44 of the P stalk, and the interaction of the mini-domain with the 40S beak. rpL12 and rpLP0 not shown.

Additional stabilization of eRF1’s interaction with the 40S subunit appears to be provided by its mini-domain, which protrudes toward the beak where it interacts via its flexible loop with h33/h34 of 18S rRNA, or possibly with the N-terminal tail of rpS31e (Figure 4).

### Interaction between eRF1 and eRF3

The rigid core of eRF1’s C-domain interacts with eRF3’s domain 3 as observed in the eRF1-eRF3_2–__3_ crystal structure (15), and with H44 of the P stalk (Figures 2 and 4). rpL12 is in proximity to eRF1’s C-domain and domain 3 of eRF3 and may contact them directly, but its scattered density suggests that they do not interact stably in this configuration. The inward rotation of the stalk base (26) is similar to that observed in Dom34/Hbs1-associated ribosomes (40).

**Figure 5.**
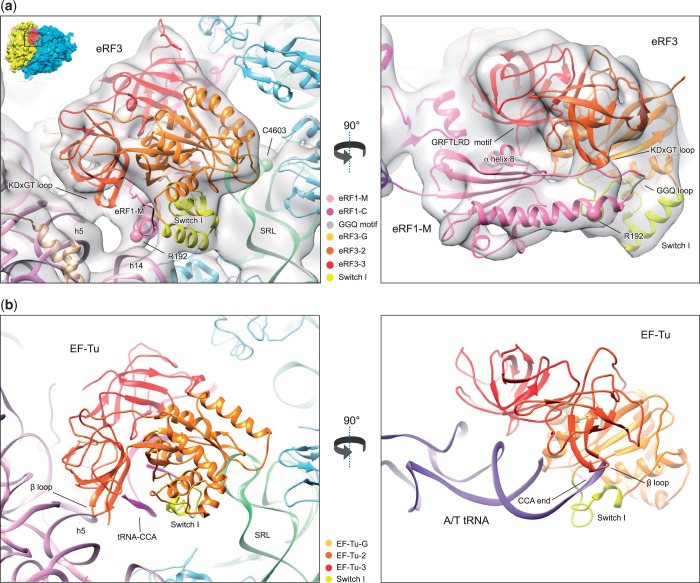
(**a**) Close-up of eRF3 showing the eRF1-eRF3 model fitted in the electron density together with the human 80S ribosome model (PDBID: 3J3A/B/D/F) [18S rRNA (purple), small subunit ribosomal proteins (beige), 28S rRNA (green), large subunit ribosomal proteins (light blue)]. (**b**) The 70S ribosome/EF-Tu/tRNA/GDPNP from (39) compared with the 80S ribosome-eRF1-eRF3 in (a).

The topology of eRF1’s M domain is now clearly visible, folded back onto eRF3, with the GGQ loop close to the switch I region, similarly to the interaction between Dom34’s central domain with Hbs1 (40) and aRF1 domain B with aEF1α (31) (Supplementary Figure S3), whereas α-helix 8 interacts with eRF3’s domain 3, close to the conserved GRFTLRD_613–__619_ motif (23) (Figure 5a).

### Structure of eRF3 bound to the ribosome

eRF3’s domains are well resolved: domains 2 and 3 have the same orientation relative to eRF1 as in the eRF1-eRF3_2–__3_ complex (15), whereas the G-domain is positioned similarly to that of aEF1α in the aRF1-aEF1α complex (31) and to G-domains in other GTPases, such as EF-Tu (39), and contacts the GAC in a similar manner (Figure 5). The switch I region of eRF3's G-domain is well ordered and is tucked between the SRL, h14 of 18S rRNA and the GGQ loop of eRF1-M. The SRL nt 4599–4600 and 4601–4603 are in proximity to DK_273–__274_ in the switch I and GE_326–__327_ in helix α5, respectively (Figure 5a).

### GTPase-activation mechanism

There are numerous similarities in the functioning of eRF1•eRF3•GTP and aa-tRNA•EF-Tu•GTP complexes that suggest likely conservation of the GTPase-activation mechanism: hydrolysis of EF-Tu/eRF3-bound GTP is triggered by binding to the A site of aa-tRNA or of eRF1, respectively, followed by dissociation of the factor and accommodation of either the aa-tRNA acceptor end or the apical loop of eRF1’s M domain in the PTC. In the case of EF-Tu, binding of aa-tRNA•EF-Tu•GTP to the ribosome induces a shift in domain 2, resulting in the interaction of a highly conserved β-turn with h5 and disruption of the interaction of the 3'-end of tRNA with the switch I loop, which leads to GTPase activation in a process that also involves direct participation of the SRL (39,53). A similar contact is established between h5 on the 40S subunit and the conserved KDxGT_449–__453_ β-turn in eRF3’s domain 2 (Figure 5a), and a C3302U substitution in the *Saccharomyces cerevisiae* SRL (equivalent to human C4603U) caused a termination defect (54). One notable difference is that there is additional density between h14 and eRF1’s M domain (Figure 5a), which involves the conserved R192 that is important for GTP hydrolysis, but not for GTP binding or the eRF1/eRF3 interaction (15). A hypothetical mechanism of GTPase activation based on the presumed similarity with EF-Tu would be that off the ribosome, eRF1’s M domain is too tightly packed onto the switch I region to allow GTP hydrolysis, but after eRF1•eRF3•GTP binds to the ribosome, the R192/h14 and β-turn/h5 interactions together pull the M-domain from eRF3, loosening the switch I region. The P stalk may act as a gatekeeper in this regard, impairing the eRF1-M/h14 and eRF3/h5 interactions until eRF1’s binding to a *bona fide* stop codon is strong enough to pull the P stalk inward. Although eRF1 can stimulate ribosome-dependent GTPase activity in the absence of an A-site stop codon (6), it is likely that GTP hydrolysis would be accelerated by the interaction between eRF1 and a stop codon.

**Figure 6.**
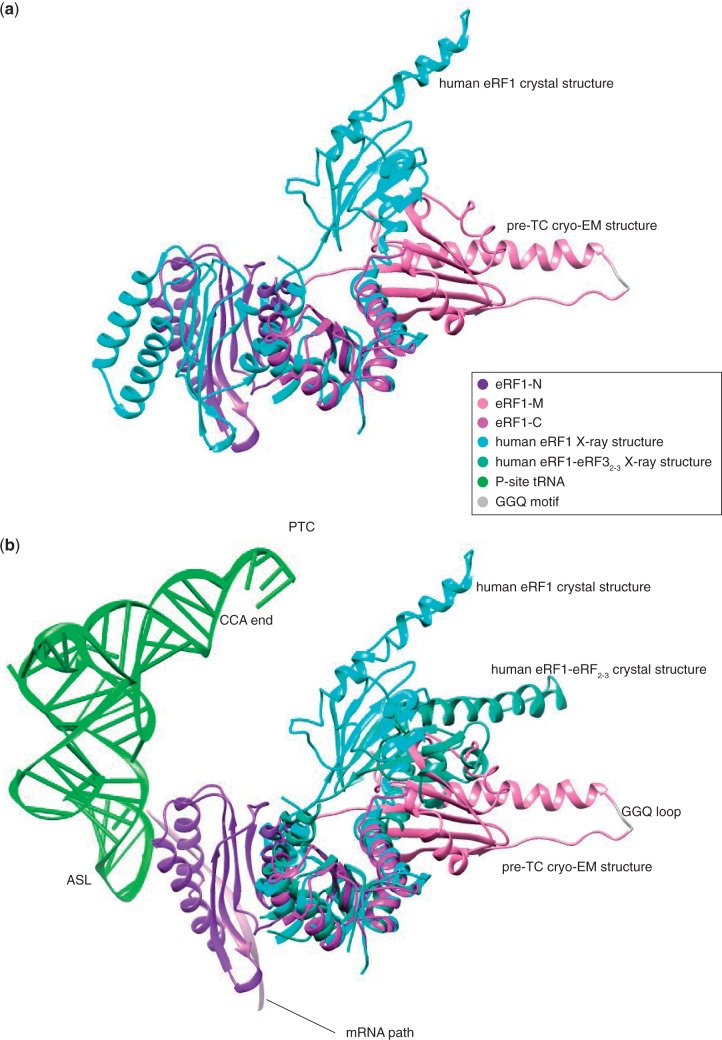
(**a**) Superimposition onto domain C of eRF1 in the crystal structure (7) (PDBID: 1DT9) and bound to the pre-TC ribosome. The mini-domain is not displayed for clarity. (**b**) Comparison between the positions of eRF1-M in the eRF1-eRF3-ribosome structure, the eRF1-eRF3_2–3_ (15) (PDBID: 3E1Y) and the eRF1 (7) (PDBID: 1DT9) crystal structures with their domain C superimposed. Position of the P-site tRNA in the context of the pre-termination complex is shown to compare the distance necessary to be travelled for the GGQ loop to reach the peptidyl transfer center (PTC) with the amplitude of movement between the different conformations of eRF1 observed. Only the N domain of eRF1 in the pre-TC cryo-EM structure is shown for clarity.

In eRF1•eRF3•GDPNP-bound pre-TCs, the GGQ loop of eRF1 is positioned far away from the PTC, and its accommodation in the PTC would require substantial rearrangement of eRF1’s domain M. Superimposing the structure of eRF1 in the eRF1•eRF3•GDPNP-bound pre-TC onto the crystal structures of individual or eRF3_2–__3_-bound eRF1 (7,15) shows that domain M can undergo large hinge movements (Figure 6). If eRF3-bound eRF1 adopts a strained conformation on binding to the ribosome similar to that of the A/T state of EF-Tu-bound tRNA (55,56), then dissociation of eRF3 following GTP hydrolysis would initiate spontaneous relaxation of eRF1 resulting in accommodation of the GGQ loop in the PTC. Full entry of the GGQ loop into the PTC would then likely be facilitated by the high mobility of the apical GGQ loop in the thermal environment (57), the positive charge of this region promoting its retention in the PTC following entry.

## CONCLUSION

Our structure revealed the topology of the interaction between eRF1’s N-domain and the UAA stop codon, as well as the intricate network of interactions between eRF3’s switch I region, eRF1’s GGQ loop and the GAC. It suggests that GTP hydrolysis may be triggered once eRF1 is properly bound to the STOP codon in a manner similar to that of EF-Tu. Now, atomic resolution structures of this complex bound to the three different stop codons may be required to achieve complete understanding of how a single factor can recognize them all.

## ACCESSION NUMBERS

The Cryo-EM map is deposited in the EMDataBank with accession code EMD-5801. The atomic model of eRF1-eRF3 bound to the pre-TC is deposited in the Protein Data Bank with the accession code 3J5Y.

## SUPPLEMENTARY DATA

Supplementary Data are available at NAR Online.

## FUNDING

Howard Hughes Medical Institute and the National Institute of Health [R01 GM29169 to J.F., R01 GM80623 to T.V.P.]. Funding for open access charge: National Institutes of Health.

*Conflict of interest statement*. None declared.

## Supplementary Material

Supplementary Data
