# Supplementary material for: Structure of the mammalian ribosomal pre-termination complex associated with eRF1•eRF3•GDPNP
Source: Nucleic Acids Res. 2013 Dec 11;42(5):3409–18. doi: 10.1093/nar/gkt1279 (PMC3950680; doi:10.1093/nar/gkt1279)
Supplement: Supplementary Data [file supp_42_5_3409__index.html]

Structure of the mammalian ribosomal pre-termination complex associated with eRF1•eRF3•GDPNP — Structure of the mammalian ribosomal pre-termination complex associated with eRF1•eRF3•GDPNP — Supplementary Data 

# Structure of the mammalian ribosomal pre-termination complex associated with eRF1•eRF3•GDPNP

## Supplementary Data

files

**Files in this Data Supplement:**

- Supplementary Data - pdf file
