# Supplementary material for: Structure of the mammalian ribosomal pre-termination complex associated with eRF1•eRF3•GDPNP
Source: Nucleic Acids Res. 2013 Dec 11;42(5):3409–18. doi: 10.1093/nar/gkt1279 (PMC3950680; doi:10.1093/nar/gkt1279)
Supplement: Supplementary Data [file supp_gkt1279_nar-02995-r-2013-File008.pdf]

SUPPLEMENTARY INFORMATION

Structure of the mammalian ribosomal pre-termination complex associated with eRF1•eRF3•GDPNP.

Amédée des Georges[1,2,] Yaser Hashem[1,2], Anett Unbehaun[3], Robert A. Grassucci[1,2], Derek Taylor[4], Christopher U. T. Hellen[3], Tatyana V. Pestova[3+] and Joachim Frank[1,2,5,+].

Affiliation: [1]HHMI, [2]Department of Biochemistry and Molecular Biophysics, Columbia University, New York, NY, USA. [3]Department of Cell Biology, SUNY Downstate Medical Center, Brooklyn, NY, USA. [4]Department of Pharmacology, Case Western Reserve University, Cleveland, OH, USA. [5]Department of Biological Sciences, Columbia University, New York, NY, USA

Correspondence should be adressed to:

Tatyana V. Pestova, SUNY Downstate Medical Center, Department of Cell Biology, 450 Clarkson Avenue, Brooklyn, NY 11203, USA.
Phone: +1 718 270-1034, Fax: +1 718-270-2656, E-mail: Tatyana.Pestova@downstate.edu

Joachim Frank, Howard Hughes Medical Institute, Department of Biochemistry and Molecular Biophysics and
Department of Biological Sciences, Columbia University, 650 W 168th Street BB2-221, New York, NY 10032, USA.
Phone: +1 212 305-9510, Fax: +1 212 305 9500, E-mail: jf2192@columbia.edu

This PDF includes:

Table S1
Figures S1-3

RELION Mode	Autorefine	Classification	Classification
HealpixOrder (angular step in degrees)	5 (1.8°)	5 (1.8°)	6 (0.9°)
OverallAccuracyRotations (degrees)	1.93°	1.13°	1.01°
OverallAccuracyTranslations (pixels)	1.53	0.923	0.865
AveragePmax	0.16	0.46	0.14

Table S1. Refinement statistics output from RELION. Particle alignment statistics output from the last iteration of each mode. Alignment accuracy and Average Pmax values are calculated according to (26). A healpix order of 5 corresponds to an angular sampling of 1.8° of the 3D sphere. A Healpix order of 6 corresponds to an angular sampling of 0.9°. The last iteration of the autorefine procedure was at healpix order 5. Its statistics are compared with the last iteration of classification mode at Healpix order 5, and with the last iteration of classification mode at healpix order 6.

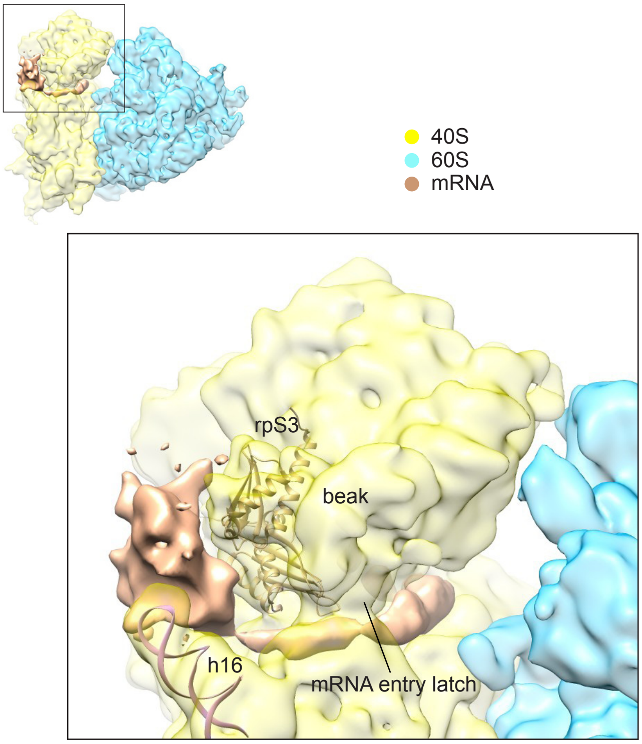

Figure S1. View of the segmented mRNA density, with the models of h16 and rpS3.

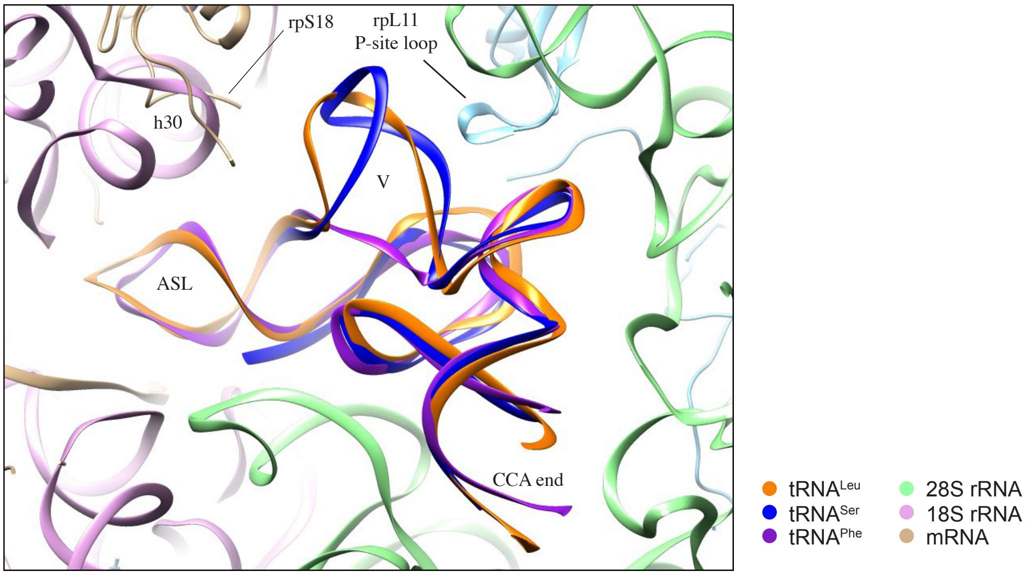

Figure S2. Potential interactions of variable loops of P site tRNAs with the ribosome. tRNAs with different variable loops superimposed: $tRNA^{Leu}$ (orange; PDBID: 1WZ2), $tRNA^{Ser}$ (dark blue; PDBID: 1SER), $tRNA^{Phe}$ (purple; PDBID: 1TTT). ASL: Anticodon Stem Loop, V: Variable region. Human 80S ribosome model rigid body docked into the density, with 18S rRNA in light pink (PDBID: 3J3D), 40S ribosomal proteins in beige (PDBID: 3J3A), 28S rRNA in light green (PDBID: 3J3F), and 60S ribosomal proteins in light blue (PDBID: 3J3B). In contrast to variable loops of conventional length (as in e.g. $tRNA^{Phe}$), long variable loops of $tRNA^{Leu}$ and $tRNA^{Ser}$ have a potential to establish contacts with the P-site loop of rpL11 on the 60S subunit and to h30 and rpS18 of the 40S subunit.

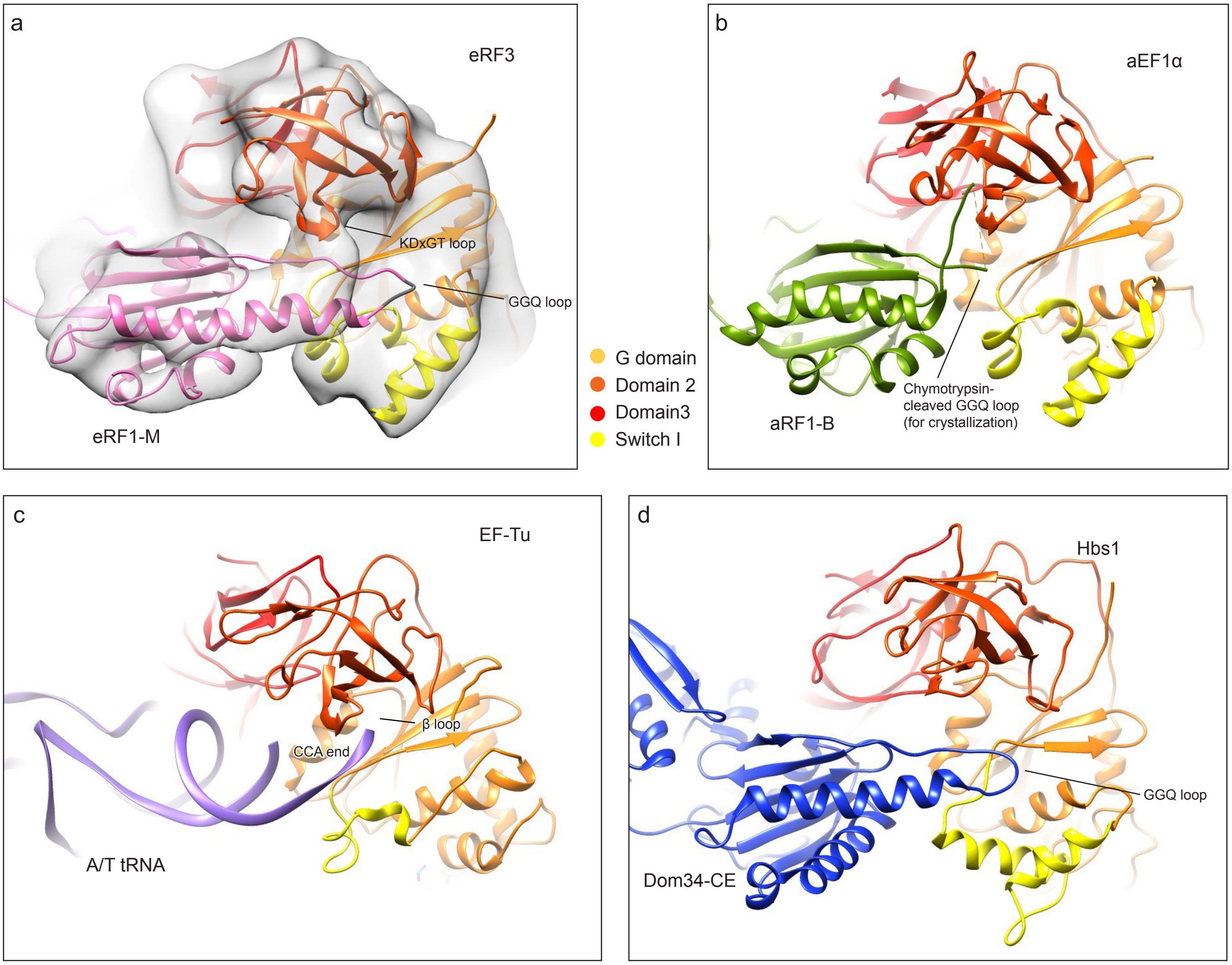

Figure S3. Comparison between the eRF1-M/eRF3 interaction and the equivalent in aRF1/aEF1α, EF-Tu/tRNA and Dom34-Hbs1. (a) eRF1-M/eRF3 (orange). **(b)** aRF1-B/aEF1α (30) (PDBID: 3VMF). **(c)** A/T tRNA/EF-Tu (38) (PDBID: 2XQD). **(d)** Dom34-central domain (Dom34-ce)/Hbs1 (39) (PDBID: 3IZQ).
